# Association of Gut Microbiota With Performance Level Among Iranian Professional and Semi‐Professional Runners: A Cross‐Sectional Study

**DOI:** 10.1002/hsr2.71319

**Published:** 2025-10-03

**Authors:** Hiwa Nazari, Armitasadat Emami Meibodi, Minoo Bassami, Meysam Olfatifar, Abbas Yadegar

**Affiliations:** ^1^ Faculty of Physical Education and Sport Sciences Allameh Tabataba'i University Tehran Iran; ^2^ Foodborne and Waterborne Diseases Research Center, Research Institute for Gastroenterology and Liver Diseases Shahid Beheshti University of Medical Sciences Tehran Iran; ^3^ Basic and Molecular Epidemiology of Gastrointestinal Disorders Research Center, Research Institute for Gastroenterology and Liver Diseases Shahid Beheshti University of Medical Sciences Tehran Iran; ^4^ Gastroenterology and Liver Diseases Research Center, Research Institute for Gastroenterology and Liver Diseases Shahid Beheshti University of Medical Sciences Tehran Iran

**Keywords:** *Akkermansia muciniphila*, athlete microbiome, *Fusobacterium nucleatum*, microbiota diversity, RT‐qPCR

## Abstract

**Background and Aims:**

The gut microbiota is a diverse ecosystem with the potential to significantly enhance athletic metabolic capacity. Emerging research indicates that the gut microbiota plays a crucial role in modulating energy metabolism, immune function, oxidative stress, skeletal muscle dynamics, and neuroendocrine regulation, all of which are essential for optimizing athletic performance. This study investigates the composition of a selection of gut microbiota among Iranian professional and semi‐professional runners from three different disciplines including endurance, middle‐distance, and speed runners, and examines their association with performance levels.

**Methods:**

Fresh stool samples of 60 runners were collected and the relative abundance of a selection of intestinal microbiota at various taxonomic levels was assessed by RT‐qPCR. The relative abundance of the selected microbiota revealed distinct patterns across different types of runners.

**Results:**

Predominant taxa in professional groups were Bacteroidetes, Firmicutes, and *Prevotella* spp., while semi‐professional groups had a higher abundance of Firmicutes, Bacteroidetes, Actinobacteria, Clostridia, and *Prevotella* spp. *Akkermansia muciniphila* was mostly abundant among speed runners (40.95%), followed by endurance (27.025%) and middle‐distance runners (23.525%). *Fusobacterium nucleatum* was more abundant in middle‐distance (34.9%) and endurance runners (34.3%) compared to speed runners (22.3%). A negative correlation was found between performance levels and the abundance of Actinobacteria, Enterobacteriaceae, E‐proteobacteria, *Bifidobacterium* spp., and *Faecalibacterium prausnitzii*, while a positive correlation was observed with *Methanobrevibacter smithii*.

**Conclusion:**

This study illuminates the distinct microbial taxa detected in professional and semi‐professional runners, which corroborates the relationship between running disciplines and the gut microbiota composition, as well as their impact on performance levels.

## 1 Introduction

The human gut microbiota is a densely inhabited ecosystem of diverse microorganisms composed of bacteria, archaea, eukaryotes, fungi, and viruses [1]. These microbes produce a variety of products, granting a broad metabolic capacity to promote the host's metabolic health, affecting energy synthesis and regulation, physiology, and fitness [2, 3]. The close interaction between the microbiota and the human host exhibits a tangible homeostasis that can be easily disrupted by intrinsic or environmental influences, a condition termed dysbiosis, potentially leading to severe host disorders [4]. Notably, studies on large population‐based cohorts have stated the substantial interindividual microbial diversity present in human populations [5]. However, the composition and metabolic activity of the gut microbial communities are influenced by a multifactorial array of elements, including diet [6], demographics [7], medication usage [3], health status [2], and various environmental agents [8]. Additionally, health behaviors, specifically physical activity and sedentary lifestyle, have been associated with considerable alterations in gut microbiota composition and function [9, 10]. Notably, the potential of the gut microbiota to respond appropriately to environmental variations is robust up to a certain threshold, beyond which its adaptive capacity is compromised [4].

A strong body of evidence suggests that the composition and diversity of the gut microbiota are highly influenced by the host's exercise patterns, depending on the type of sport as well as the intensity of the activity [11, 12, 13]. Research conducted through cross‐sectional studies has indicated that leading a physically active lifestyle or participating in moderate exercise can enhance gut microbiota diversity and stimulate the growth of beneficial microbes [14]. Moreover, the average time spent exercising during a week and athletes' performance level can affect the composition of the gut microbiome [15]. The impact of gut microbiota on physiological and metabolic adaptations, such as enhanced muscle strength, high aerobic capacity, increased energy expenditure, and heat production, significantly influences the performance of professional athletes [16]. In addition, the gut microbiota plays an important part in modulating the host immune system and the integrity of mucosal membranes, thus influencing brain health, which can affect the performance of sportsmen [17]. Athletes' microbiota can play a key role in fermenting the colon organic compounds leading to the production of bacterial by‐products such as short‐chain fatty acids (SCFAs), which are absorbed and exploited by host cells [18]. Moreover, the beneficial microbiota can consume lactate produced during exercise, which theoretically may reduce postexercise recovery time and even affect physical performance [17, 19]. Therefore, understanding the various functions of gut microbiota in athletic performance has been of great interest in recent years, particularly for improving competition outcomes and enhancing athletic capacity during competitions [20, 21, 22].

At present, research focusing on the microbiome profile of athletes in different disciplines and races remains confined [23]. Given the differences in the intensity and volume of training in runners of various disciplines, as well as the distinctive energy systems they utilize, each type of runner can possess unique physiological characteristics [24, 25]. Accordingly, this study aimed to investigate the relative abundance of a selection of bacterial taxa among speed, middle‐distance, and endurance runners, and to examine the correlation between these changes and performance levels in Iranian runners.

## 2 Methods

### 2.1 Subject Recruitment and Experimental Design

**Figure 1:**
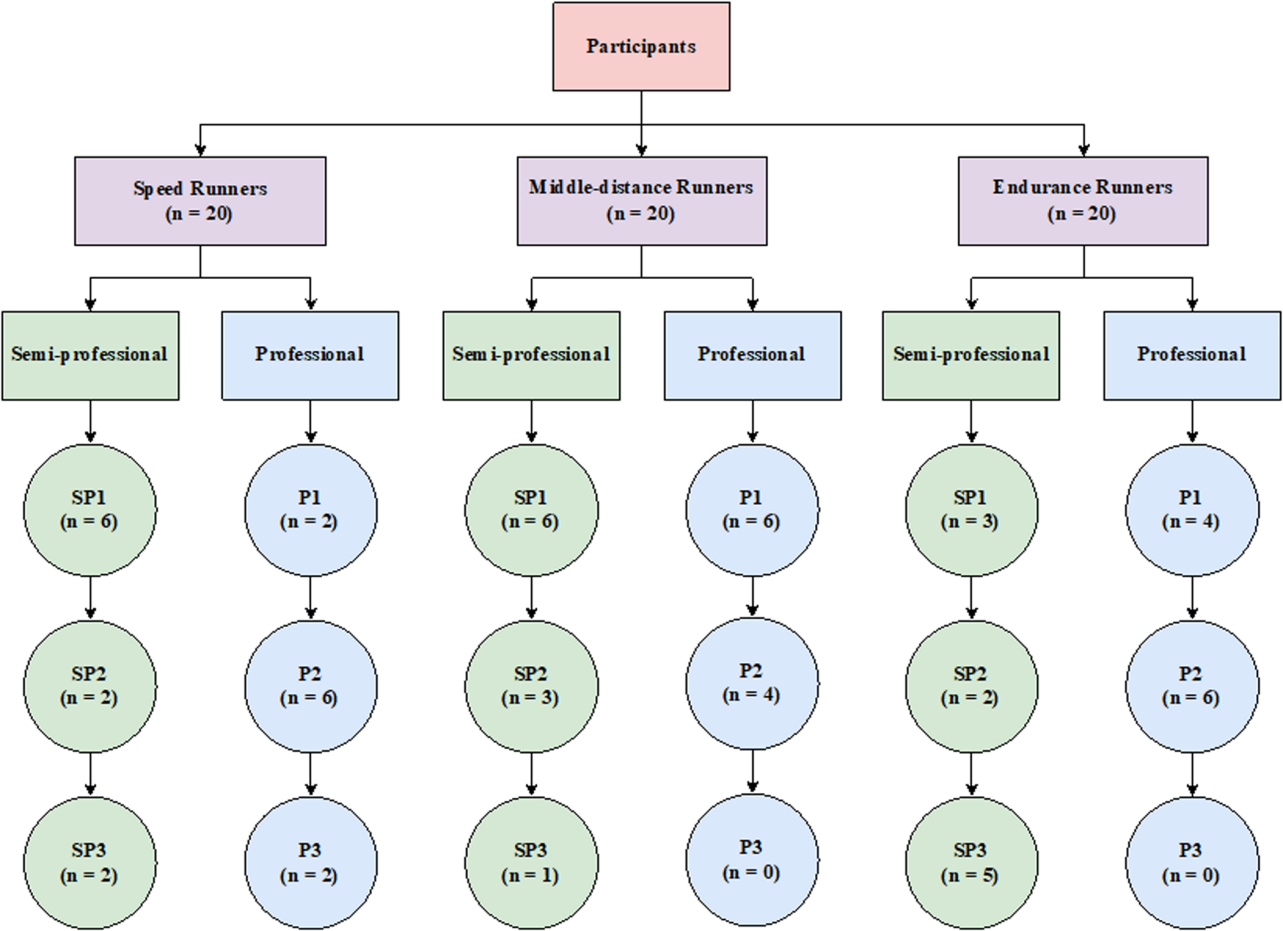
Recruitment of participants. Each category reflects the range of experience among the study cohort. Sp1, provincial medal; Sp2, student Olympiad medal; Sp3, premier league participates; P1, national medal; P2, Asian medal; P3, Tokyo 2020 Olympics participate.

The intestinal microbiome of 60 selected runners in three different types of training disciplines, including endurance runners (5000 m and 10000 m, *n* = 20), middle‐distance runners (800 m and 1500 m, *n* = 20), and speed runners (100 m, 200 m 400 m, *n* = 20) were investigated (Figure 1). As part of the exclusion criteria, factors such as medication intake within the preceding 3 months, alcohol consumption, and pre‐existing gastrointestinal or systemic diseases were carefully controlled to make sure they did not influence the outcomes. The study cohort consisted of runners with a minimum of 3 years of semi‐professional training and at least 5 years of professional running experience. Participants were classified into performance levels based on their competitive history. This classification was verified using official athletic records provided by the Athletics Federation of I.R Iran. The semi‐professional participants were categorized into three groups: Sp1, comprising those with provincial medals; Sp2, including those who had medaled in student Olympiads; and Sp3, featuring individuals who had participated in premier leagues. The professional participants were similarly divided into three groups: P1, consisting of runners with national medals; P2, those with Asian medals; and P3, individuals who had competed in the Tokyo 2020 Olympics. This study was conducted in accordance with the principles outlined in the Declaration of Helsinki and adhered to CONSORT reporting guidelines where applicable. Written informed consent was obtained from all participants before enrollment. The study was approved by the Ethical Review Committee of Allameh Tabataba'i University (Project No: IR.ATU.REC.139.054). The selection of runners and subsequent experiments conducted in this study adhered strictly to the relevant guidelines and regulations recommended by the aforementioned committee. Written informed consent was obtained from all subjects before sample collection. All participants filled out a questionnaire covering their diet, 3‐day food recalls, and average weekly exercise hours. Dietary profiles were categorized based on participant‐reported food recall data into groups including high complex carbohydrates, balanced macronutrient intake (equal protein, fat, and carbohydrates), high protein, vegetarian, and gluten‐free (Supporting Information Tables S1–S3).

### 2.2 Fecal Sample Collection and DNA Extraction

Fresh stool samples were collected from all participants in sterile collection containers during a 4‐week frame in August 2024, following an overnight fast of at least 8 h. To minimize diurnal and dietary variability in microbial composition, all samples were obtained in the early morning hours, between 6:00 to 9:00 AM, before breakfast. Immediately after voiding, samples were placed on frozen freezer packs and transported to the Research Institute for Gastroenterology and Liver Diseases, Shahid Beheshti University of Medical Sciences, Tehran, Iran. Each sample was homogenized by vortexing and divided into aliquots within 4 h of collection. The aliquots were immediately frozen and stored at − 80°C in screw‐capped cryovial tubes until DNA extraction.

### 2.3 Gut Microbiota Profiling by Quantitative Real‐Time PCR

Quantification of different microbiota phyla, classes, and genera was carried out using a Rotor‐Gene Q (Qiagen, Hilden, Germany). All amplifications were performed in triplicate in a final volume of 20 µl containing 10 µl of a 2x SYBR green Master Mix (BIOFACT CO. Ltd., Daejeon, South Korea) including 10 pmol of each primer, and 50 ng of DNA template. Primer specificity was confirmed through melting curve analysis, where a single peak without primer‐dimer formation was observed. Additionally, standard curves were generated using 10‐fold serial dilutions of purified genomic DNA from reference bacterial strains to determine the amplification efficiency of each primer pair. Amplification efficiency (E) was calculated using the slope of the standard curve based on the formula: E (%) = [10^(−1/slope) − 1] × 100. The efficiency values for both universal (Eubacteria) and taxon‐specific primers ranged between 91% and 98%, which met the acceptable threshold for reliable quantification. Only primers with R² values ≥ 0.98 and specific melting temperatures (Tm) were included in the analysis. The amplification was performed with an initial DNA denaturation step at 95°C for 15 min; followed by 40 cycles at 95°C for 20 s; primer annealing at 56°C for 30 s; extension at 72°C for 20 s. The average Ct value obtained from each reaction was transformed into the following percentage formula:

$$x=\frac{{\left(\text{EFF}.\text{Univ}\right)}^{Ct\;univ}}{{\left(\text{EFF}.\text{Spec}\right)}^{Ct\;spec}}\times 100.$$

The Eff. Univ refers to the calculated efficiency of the universal primers for Eubacteria (2 = 100% and 1 = 0%) and Eff. Spec indicates the efficiency of the taxon‐specific primers. Ct univ and Ct spec represent the threshold cycles registered by the thermocycler. X addresses the percentage (%) of taxon‐specific 16S rRNA gene copy numbers in an individual fecal sample. The microbiota‐targeted primers used in this study are listed in Table 1. The relative abundance of each bacterial taxon was normalized against the total bacterial 16S rRNA gene content using universal Eubacteria primers as a reference. The normalization approach could express taxon‐specific abundance as a percentage of the total bacterial community within each sample. Calculations incorporated the respective amplification efficiencies of universal and taxon‐specific primers to ensure accurate quantification [26].

**Table 1:** Primers used for RT‐qPCR assays.

Target gene	Oligonucleotide sequence (5'−3')	Size (bp)	TM (°C)	References
Eubacteria	ACTCCTACGGGAGGCAGCAGT	~200	64	27
	ATTACCGCGGCTGCTGGC			
Firmicute	GGAG**Y**ATGTGGTTTAATTCGAAGCA AGCTGACGACAACCATGCAC	~129	66	1
Bacteroidetes	GTTTAATTCGATGATACGCG TTAAGCCGACACCTCACG	~137	56	1
Actinobacteria	AAATGACGGTACCTGACTA CTTTGAGTTTCATTCTTGCGA	~333	56	28
Ε‐proteobacteria	TGGTGTAGGGGTAAAATCCG AGGTAAGGTTCTTCG**Y**GTATC	~286	60	29
Clostridia	AAAGGAAGATTAATACCGCATA	~538	57	30
	TTCTTCCTAATCTCTACGCA			
Enterobacteriaceae	CATTGACGTTACCCGCAGAAGAAGC	~195	66	31
	CTCTACGAGACTCAAGCTTGC			
*Veillonella* spp.	ACCAACCTGCCCTTCAGA	~110	58	32
	CGTCCCGATTAACAGAGCTT			
*Lactobacillus* spp.	TGGATGCCTTGGCACTAG AAATCTCCGGATCAAAGCTTAC	~89	64	33
*Bifidobacterium* spp.	GGGATGCTGGTGTGGAAGAG	~200	64	34
	TGCTCGCGTCCACTATCCAG			
*Prevotella* spp.	CACCAAGGCGACGATCA	~507	58	32
	GGATAACGCCTGGACCT			
*Clostridium coccoides* group	AAATGACGGTACCTGACTA	~438	56	35
	CTTTGAGTTTCATTCTTGCGA			
*Akkermansia mucinphila*	CAGCACGTGAAGGTGGGGAC	~329	64	36
	CCTTGCGGTTGGCTTCAGAT			
*Faecalibacterium prausnitzii*	GATGGCCTCGCGTCCGATTAG	~198	66	32
	CCGAAGACCTTCTTCCTCC			
*Fusobacterium nucleatum*	GATCCAGCAATTCTGTGTG	~290	58	32
	CGAATTTCACCTCTACACTTG			
*Methanobrevibacter smithii*	CGATGCGGACTTGGTGTTG	~184	62	32
	TGTCGCCTCTGGTGAGATGTC			

*Note:* The nucleotides in bold type represent: Y, C or T.

### 2.4 Statistical Analysis

All analyzes were performed using R (version 4.x) and SPSS version 21.0 (IBM Corp.). R packages used included tidyverse, readxl, ggpubr, and boot. A multivariate analysis of variance (MANOVA) was performed to assess differences in dietary variables. Wilks' lambda and associated F‐tests were used to evaluate group differences across the multivariate dietary profile. Based on normality, parametric tests (t‐test, one‐way ANOVA, followed by Tukey's post hoc test) or their non‐parametric equivalents (Mann‐Whitney U test, Kruskal‐Wallis test, followed by Dunn's post hoc test with Bonferroni correction) were applied. Correlation between microbial taxa and performance level was assessed using Pearson or Spearman correlation coefficients, depending on data distribution, and corresponding *p* value and 95% confidence intervals (CIs) were reported. For all correlation analyzes, the False Discovery Rate (FDR) method was applied to adjust for multiple comparisons. FDR‐adjusted *p* values < 0.05 were considered statistically significant. Results are presented as mean ± standard deviation (SD) or median with interquartile range (IQR), as applicable. All tests were two‐sided, and a *p* value < 0.05 was considered statistically significant. Figures were generated using the ggplot2 package in R version 3.6.1 (R Core Team, Vienna, Austria). Analyzes related to gut microbiota differences by runner group were pre‐specified, whereas correlations between taxa and performance metrics were considered exploratory. This statistical reporting approach follows the recommendations by Assel et al. and SAMPL guidelines [27, 28].

## 3 Results

### 3.1 Anthropometric Characteristics of Runners

The anthropometric characteristics of the participants are summarized in Table 2. Sixty runners were included in the study (Figure 1), divided equally into three groups: speed runners (*n* = 20), middle‐distance runners (*n* = 20), and endurance runners (*n* = 20). The mean ages of the participants were 22.25 ± 2.69 years for speed runners, 21.73 ± 3.99 years for middle‐distance runners, and 24 ± 3.26 years for endurance runners. The mean BMI values were 22.5 kg/m² for speed runners, 21.4 kg/m² for middle‐distance runners, and 21.2 kg/m² for endurance runners. The assessment of dietary intake revealed that while dietary patterns varied across runner groups, the majority of participants across all categories reported a high intake of complex carbohydrates. A smaller proportion reported balanced macronutrient intake or high protein diets. Vegetarian and gluten‐free patterns were reported in a few cases. Notably, no statistically significant differences in dietary variables were detected between runner groups (Supporting Information Tables S1–S3).

**Table 2:** Anthropometric characteristics of the runners participated in this study.

Variables	Speed runners		Middle‐distance runners		Endurance runners	
	Mean	SD	Mean	SD	Mean	SD
Age (years)	22.25	2.69	21.73	3.99	24	3.26
Height (cm)	178.8	5.36	177.1	4.46	176.3	4.65
Weight (kg)	72.2	5.46	67.3	4.40	65.9	5.36
BMI (kg/m^2^)	22.5	—	21.4	—	21.2	—

Abbreviations: BMI, body mass index; SD, standard deviation.

### 3.2 Relative Abundance of Bacterial Taxa

**Figure 2:**
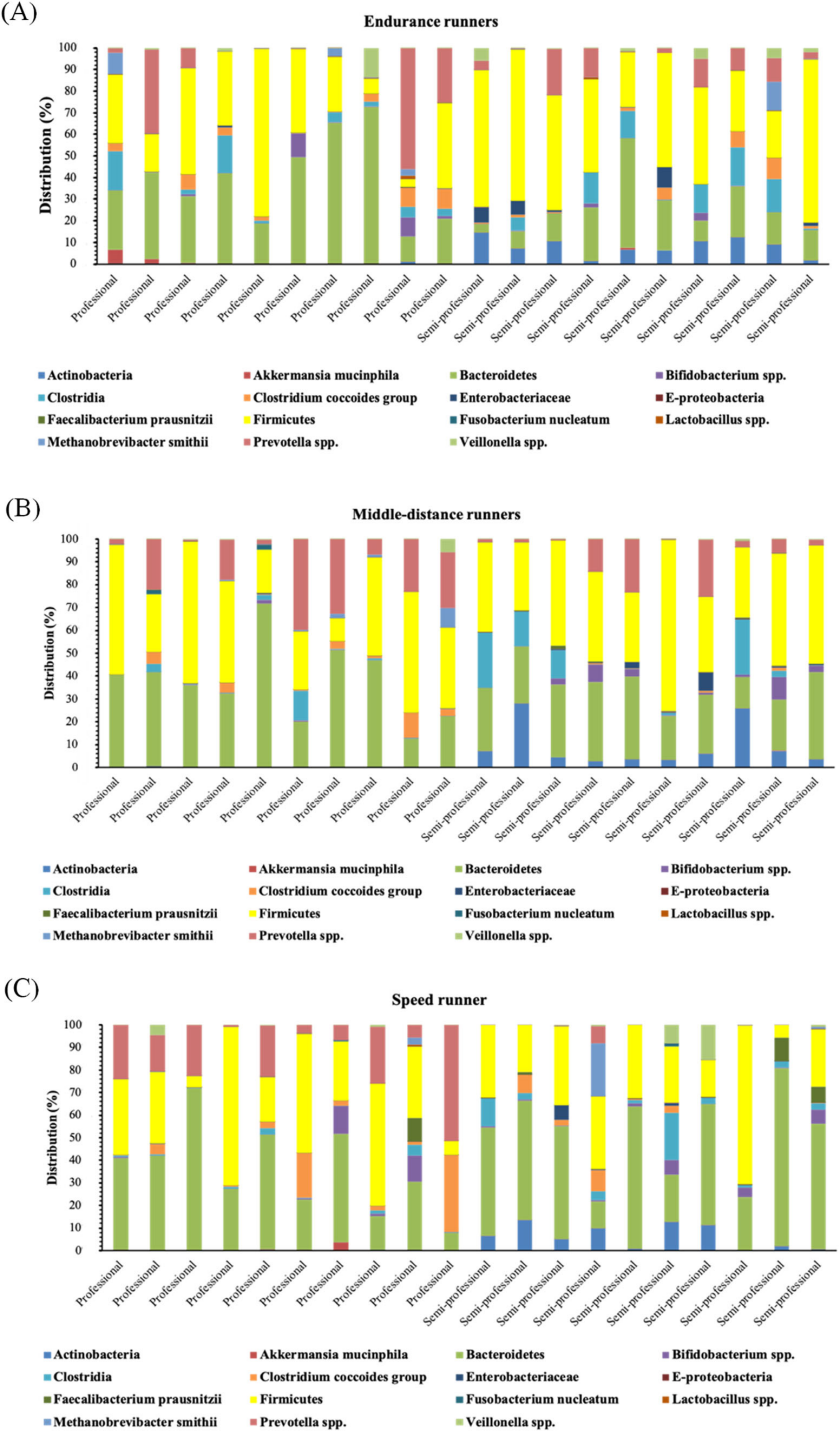
Relative abundance of selected gut microbiota among professional (*n* = 30) and semi‐professional (*n* = 30). Bar plots represent the mean relative abundance (%) of targeted gut microbial taxa in fecal samples from endurance (*n* = 20) (A), middle‐distance (*n* = 20) (B), and speed (*n* = 20) (C) runners at the phylum, class, genus, and species levels. Statistical differences were assessed using one‐way ANOVA or Kruskal‐Wallis tests, followed by Tukey's post hoc test or Dunn's test with Bonferroni correction, respectively.

Fifteen bacterial phyla, classes, and genera in the fecal samples of professional and semi‐professional runners were analyzed using RT‐qPCR. The mean percentages of the examined fecal communities across the study groups are presented in Figure 2. The mean percentage results indicated that the predominant phyla in all semi‐professional groups were Firmicutes, Bacteroidetes, Actinobacteria, Clostridia, and *Prevotella* spp., and in the professional group were Bacteroidetes, Firmicutes, and *Prevotella* spp.

### 3.3 Firmicutes to Bacteroidetes Ratio

**Figure 3:**
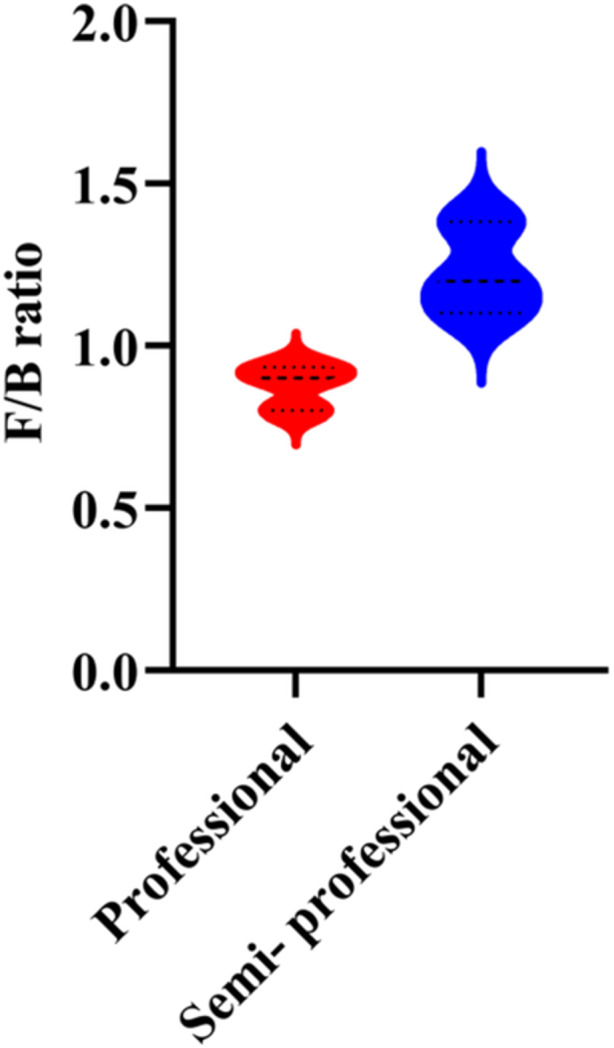
Firmicutes to Bacteroidetes (F/B) ratio in professional and semi‐professional runners. Violin plots show the F/B ratio in fecal samples from professional (*n* = 30) and semi‐professional (*n* = 30) runners. The F/B ratio was calculated based on the relative abundance of each phylum as quantified by RT‐qPCR, which was normalized against total bacterial 16S rRNA gene content. Each point represents one participant (*n* = 30 per group). Statistical comparison was performed using the Mann‐Whitney U test (*p* = 0.031).

The Firmicutes/Bacteroidetes (F/B) ratio is used to determine the normal homeostasis in the gut microbiota composition. Herein, F/B ratio was calculated based on relative abundance values derived from RT‐qPCR data. We utilized F/B ratio to show the changes in targeted gut microbiota communities in fecal samples of professional and semi‐professional runners. As illustrated in Figure 3, F/B ratio was lower in the professional runners; however, the difference was not significant (*p* = 0.1).

### 3.4 The Association Between Gut Microbiota and Running Performance

**Figure 4:**
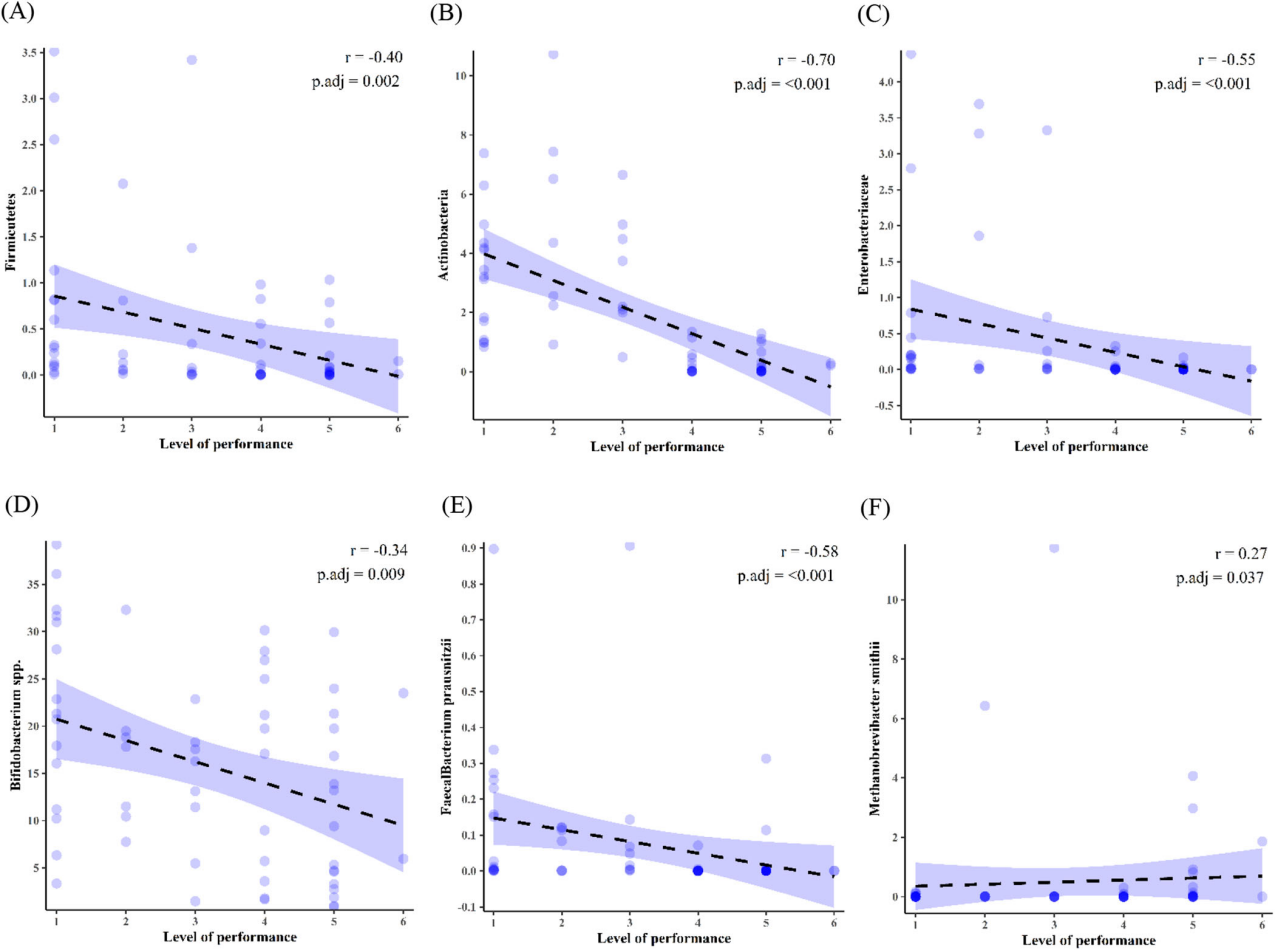
Correlation between performance level and gut microbiota abundance. A total of 60 runners were categorized into six performance levels based on officially recorded race completion times and national ranking achievements. Spearman's correlations were calculated between performance level and the relative abundance of (A) Firmicutes (r = − 0.402, *p* = 0.0015; 95% CI: − 0.599 to − 0.165), (B) Actinobacteria (r = − 0.696, *p* < 0.0001; 95% CI: − 0.775 to − 0.592), (C) Enterobacteriaceae (r = − 0.551, *p* < 0.00001; 95% CI: − 0.683 to − 0.386), (D) *Bifidobacterium* spp. (r = − 0.344, *p* = 0.0072; 95% CI: − 0.563 to − 0.061), (E) *F. prausnitzii* (r = − 0.578, *p* < 0.00001; 95% CI: − 0.705 to − 0.341), and (F) *M. smithii* (r = 0.270, *p* = 0.037; 95% CI: 0.032 to 0.471). Each data point represents an individual participant.

The relationship between gut microbiota composition and running performance was examined by separating runners into six performance levels based on race completion times, with level 1 representing the fastest and level 6 the slowest. As illustrated in Figure 4, several microbial taxa exhibited statistically significant correlations with performance level. Firmicutes demonstrated a moderate negative correlation (r = − 0.402, *p* = 0.0015; 95% CI: − 0.599 to −0 .165) with performance level. Moreover, the results revealed a negative correlation between runners' performance level and the abundance of Actinobacteria (r = − 0.696, *p* < 0.0001; 95% CI: − 0.775 to − 0.592), Enterobacteriaceae (r = − 0.551, *p* < 0.00001; 95% CI: − 0.683 to − 0.386), *Bifidobacterium* spp. (r = − 0.344, *p* = 0.0072; 95% CI: − 0.563 to − 0.061), *Faecalibacterium prausnitzii* (r = − 0.578, *p* < 0.00001; 95% CI: − 0.705 to − 0.341). Conversely, a positive, relatively weak correlation between *Methanobrevibacter smithii* (r = 0.270, *p* = 0.037; 95% CI: 0.032–0.471) and runners' performance level was observed. Correlation statistics, including Spearman's rho, 95% CI, raw *p* values, and FDR‐adjusted *p* values, are provided in Supporting Information Table S4.

### 3.5 Distribution of Akkermansia muciniphila and Fusobacterium nucleatum in Runners

**Figure 5:**
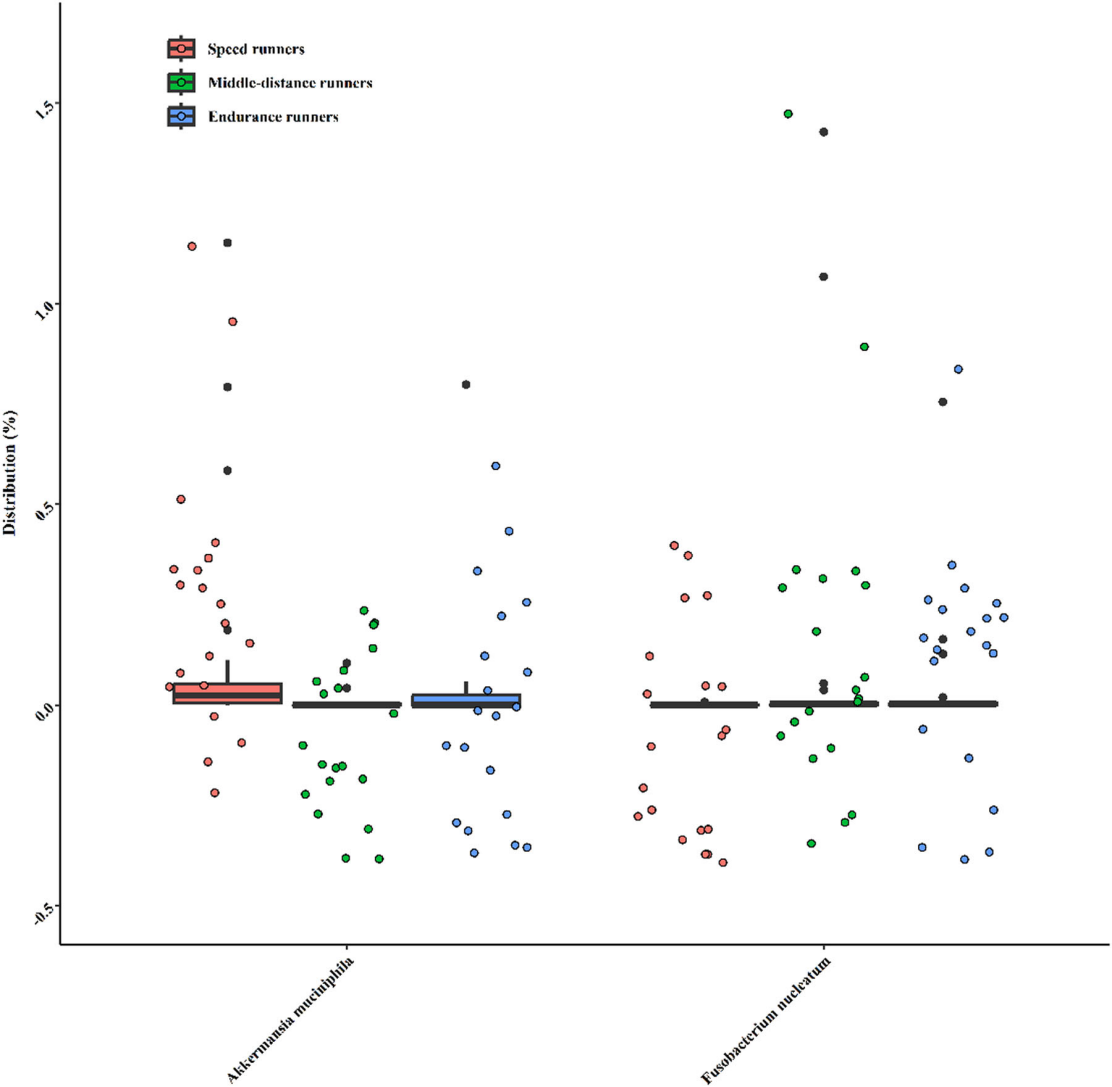
Distribution of *A. muciniphila* and *F. nucleatum* in speed (*n* = 20), middle‐distance (*n* = 20), and endurance (*n* = 20) runners. Data are presented as mean relative abundance (%), with error bars indicating standard deviation. Between‐group differences were considered significant at *p* < 0.05 and are annotated in the figure. *A. muciniphila* abundance was significantly higher in endurance runners compared to speed runners (FDR‐adjusted *p* = 0.008), while *F. nucleatum* was significantly elevated in endurance compared to middle‐distance runners (FDR‐adjusted *p* = 0.012).

The distribution of *A. muciniphila* and *F. nucleatum* was evaluated in the study groups. As shown in Figure 5, *A. muciniphila* was the most abundant species among speed runners (40.95%), followed by endurance runners (27.025%) and middle‐distance runners (23.525%). Inversely, *F. nucleatum* was more abundant in middle‐distance runners (34.9%) and endurance runners (34.3%) compared to speed runners, where it constituted 22.3% of the bacterial population.

### 3.6 The Distinct Relative Abundance of Gut Microbiota Among Different Runner Groups

**Figure 6:**
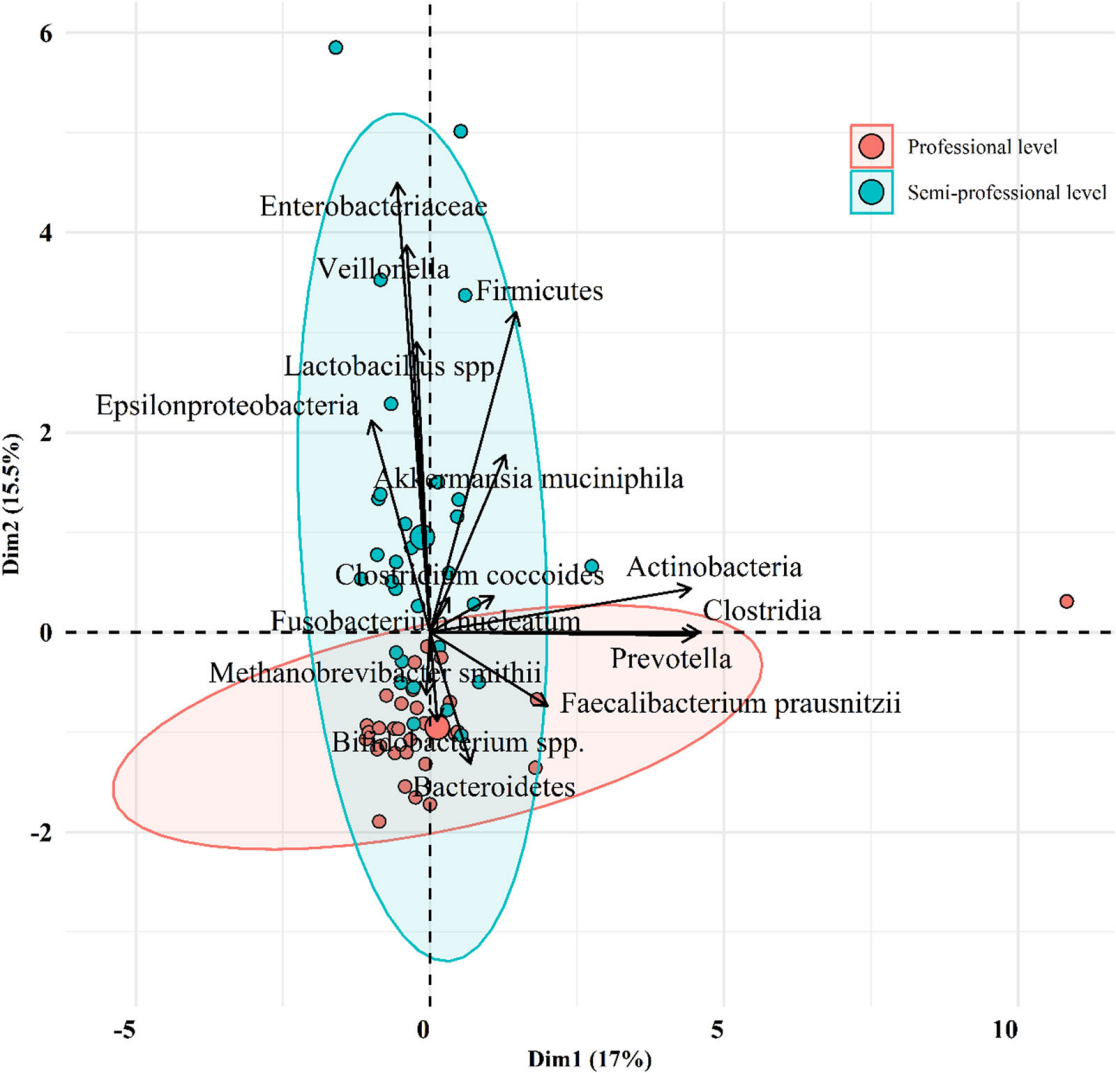
Bacterial community clustering using principal component analysis (PCA) for professional (*n* = 30) and semi‐professional (*n* = 30) groups of runners. Percentage values in parentheses next to Dim1 and Dim2 represent the percentage of variance explained by each component. Arrows show the contribution of each type of microbiota on the Dim1 and Dim2. Each data point denotes an individual runner, colored based on their group. Ellipses represent 95% confidence intervals for each runner group.

**Figure 7:**
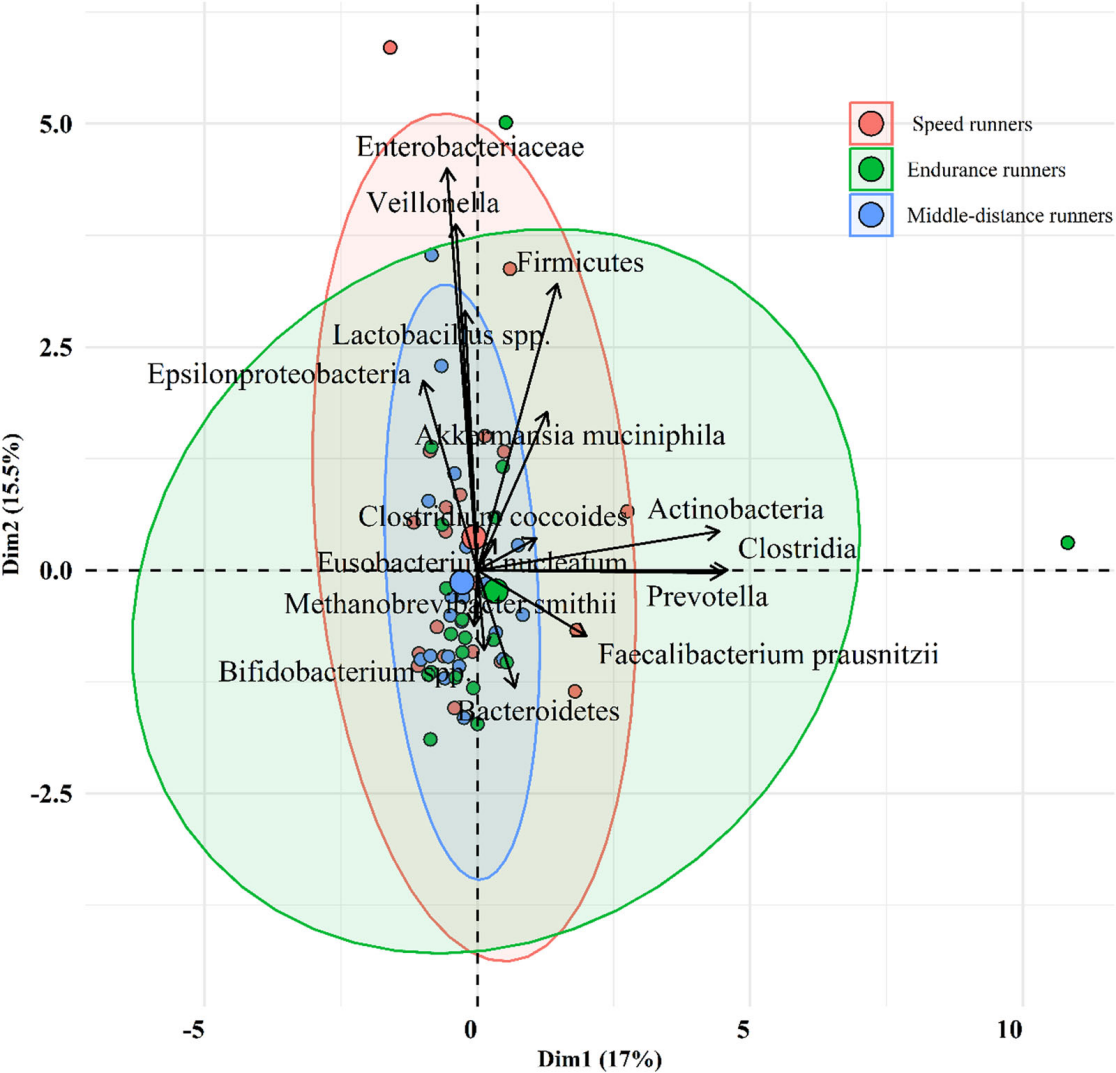
Bacterial community clustering using principal component analysis (PCA) for speed runners (*n* = 20), middle‐distance runners (*n* = 20), and endurance runners (*n* = 20). Percentage values in parentheses next to Dim1 and Dim2 represent the percentage of variance explained by each component. Arrows show the contribution of individual taxa, and shaded ellipses indicate the 95% confidence intervals for each performance group.

The results from the principal coordinate analysis (PCA) elucidated overlapping abundances of Bacteroidetes, *Clostridium coccoides* group, *Prevotella* spp., and *A. muciniphila* between professional and semi‐professional runners, however, differences have been observed (Figure 6). Within the semi‐professional group, there were elevated levels of Actinobacteria, *Epsilonproteobacteria*, *Veillonella*, Clostridia, *M. smithii*, and Firmicutes, whereas Prevotella and *Clostridium coccoides* group were elevated in the professional group. In addition, the middle‐distance runners showed increased levels of E‐proteobacteria and *Veillonella*, while the speed runners had increased levels of Actinobacteria, Firmicutes, and *M. smithii* (Figure 7).

## 4 Discussion

Physical exercise plays an important part in maintaining homeostasis and energy regulation by influencing the composition of gut microbiota [29]. Different forms of exercise with variations in frequency, mode, and intensity have been shown to instigate notable changes in gut microbial composition [30]. Exercise can accelerate the development of a robust, diverse, and complex microbiota, promoting the colonization of beneficial bacteria, which form a barrier against pathogens, protect the intestinal epithelium from infection, and enhance overall gut health [31]. Moreover, the interaction between muscle and the gut microbiota can positively impact muscle mass, quality, and function [32, 33]. Controlled trials show that microbiota composition, like butyrate‐producing taxa, not only improves muscle mass, but also reduces fat mass in athletes who receive both protein and synbiotic supplementation [22]. Diet and the level of physical activity are recognized as the principal causal factors for functional and compositional shifts within an established gut milieu [32]. This community of microorganisms has reciprocal roles in fuel selection and systemic metabolism in response to muscle contractile force during physical activity and participates in the elevation of exercise capacity [34, 35]. Additionally, microbial taxa enriched in the intestinal tract of athletes are known to be instrumental in fiber fermentation [36]. Following an exercise‐rich lifestyle, the modulated composition and metabolic capacity of the gut microbiome with a substantial production of SCFA contribute to high cardiorespiratory fitness, which in turn enhances exercise efficiency [37]. A body of evidence has linked a higher abundance of certain microbiota, like *Veillonella* or *Prevotella* to increased SCFA production and improved performance quality [38, 39]. A recent case‐control study by Humińska‐Lisowska et al. similarly reported positive correlations between *Bifidobacterium longum*, *B. adolescentis*, and higher VO_2_max in trained individuals, along with increased abundance of SCFA producers such as *Blautia wexlerae* and *Roseburia hominis*, which reinforced the link between gut‐derived metabolites and athletic capacity [40]. In our study, we also observed that as the runners' level of professionalism increased, the abundance of *Prevotella* correspondingly elevated, which has been proposed to potentially enhance their performance by improving metabolic pathways linked to energy extraction and anti‐inflammatory responses. A recent systematic review has found that *Prevotella* relative abundance was associated with training duration [41]. Another systematic review found a similar result with a high *Prevotella* abundance associated with time‐reported exercising during an average week [40]. *Prevotella* is known for its role in carbohydrate metabolism, mostly in fermenting fiber to produce SCFAs like propionate, which may serve as an energy source during prolonged exercise [42, 43]. While our findings show increased *Prevotella* abundance in professional runners, the functional consequences remain speculative and warrant confirmation through metabolomic or functional assays.

The interaction between physical activity and gut microbiota composition is a complex and multi‐factorial relationship influenced by exercise intensity, duration, and type, which is frequently associated with increased microbial diversity and Function [9]. For instance, some studies indicate that exercise increases the F/B ratio, while others suggest a decrease [38]. Furthermore, it has been shown that physical activity can upregulate the F/B ratio independently of dietary factors, thereby impacting body weight [44]. A decreased F/B ratio was associated with improvements in obesity‐related metabolic disturbances, including lipid dysregulation, inflammation, and hepatic steatosis [45]. Herein, our findings showed that F/B ratio was lower among professional runners, despite being insignificant. Of note, professional runners typically have different metabolic and physiological responses compared to the general population, which could lead to distinct changes in their gut microbiota [46]. Professional runners may experience low‐grade gut inflammation due to intense and frequent training, which can directly impact the gut microbiota composition, resulting in a reduced F/B ratio [47]. Additionally, the diet of the participants was not meticulously controlled or analyzed in this study. Diet is a crucial factor that influences gut microbiota composition, and individual differences in dietary intake could potentially affect the results [48].

Hereon, we have elucidated a positive relationship between the presence of *M. smithii* in fecal samples and the level of athletic performance among various runners in the cohort. In accordance with this study, Petersen et al. [49] performed a metatranscriptome analysis on the stool samples of a group of highly fit cyclists and showed an elevated abundance of *M. smithii* transcripts in professional cyclists in comparison to amateur ones. As a hydrogenotrophic methanogen, *M. smithii* consumes hydrogen generated by fermentative gut microbes and converts it into methane via methanogenesis. This process alleviates feedback inhibition on bacterial fermentation through reducing hydrogen partial pressure in the distal colon to enhance the breakdown and utilization of complex polysaccharides [50, 51, 52]. *M. smithii* can maintain an optimal redox balance in the gut via removing excess hydrogen to favor increased production of SCFAs, which are readily absorbed and utilized by the host as key sources of energy. SCFAs such as acetate, propionate, and butyrate can be absorbed by colonocytes and hepatocytes, where they enter host metabolic pathways, including the citric acid cycle and gluconeogenesis, ultimately contributing to systemic ATP production [53]. It has been previously indicated that a higher abundance of *M. smithii* in professional athletes suggests a potential role for this species in promoting interspecies hydrogen transfer and enhancing energy substrate availability through microbial fermentation [54]. Accordingly, *M. smithii* may contribute to promoting syntrophic interactions and potentially translate into energetic resources for high‐performance individuals. While we observed a positive association between *M. smithii* and performance, this mechanistic link remains hypothetical in the absence of direct metabolite measurements. In this regard, Petersen et al. [49] believed that *M. smithii* may also participate in the reduction of recovery time from vigorous aerobic activities and may even influence race performance.

Our further investigations on the relationship between the gut microbiota and the quality level of performance in running showed a negative correlation between Actinobacteria, E‐proteobacteria, Enterobacteriaceae, *Bifidobacterium* spp., and *F. prausnitzii*, and performance level. Of note, members of E‐proteobacteria, such as *Helicobacter pylori*, have been linked to gastrointestinal discomfort, poor quality of life, and low skeletal muscle mass [55, 56]. Using RT‐qPCR assay and the Illumina MiSeq platform, Evans et al. [57] investigated the gut microbial composition of mice subjected to high‐fat and low‐fat diets following voluntary exercise. Their findings showed notable parallels to our study on humans, despite the inherent differences between the two species. They reported that wheel running in low‐fat‐fed mice resulted in a decrease in Actinobacteria, the order Bifidobacteriales, and the family Bifidobacteriaceae. In addition, Lambert et al. [58] used RT‐qPCR to assess cecal samples of type 2 diabetic and normal mice after 6 weeks of being sedentary or physically active. Despite the unchanged amount of Enterobacteriaceae in controls, the cecal abundance of this family of bacteria decreased in diabetic mice post‐intervention compared to baseline. Notwithstanding the lower abundance of *Bifidobacterium* spp. in exercised diabetic mice, this family was significantly detected in healthy controls after the course of physical activity. Besides, the supplementation of a *Bifidobacterium longum* strain with probiotic properties, which has been isolated from a weightlifting Olympic champion, alongside endurance events in mice resulted in elevated athletic performance and improved physiological adaption [59].

The comparison of the gut microbiota between two groups of elderlies, including community‐dwelling older adults and physically active senior orienteering athletes has indicated that the physically active seniors harbor a more homogeneous microbiota with an increased abundance of *F. prausnitzii* compared to the other elderlies [60]. *F. prausnitzii* is one of the pivotal butyrate producers in the human colon with a vital role in providing energy for the colonocytes, the reduction of which has been associated with the onset of inflammatory processes [61]. It has been demonstrated that an increase in *F. prausnitzii* and *A. muciniphila* in high‐performing athletes may support anti‐inflammatory and energy regulatory effects, yet the underlying mechanisms remain speculative without concurrent SCFA quantification [20]. Although we found a negative association between *F. prausnitzii* abundance and performance level, the inflammatory status of participants was not evaluated. Notably, the effect of sportive activities on intestinal health is believed to be a hormetic‐shaped curve with moderate exercise improving leaky gut and inflammatory response, contrary to drastic enduring activities that disrupt the gut barrier [62]. As a result, endurance sportsmen often deal with various gastrointestinal symptoms, like abdominal cramps, nausea, and diarrhea, during or after practice [63]. It has been elucidated that intense bouts of exercise give rise to the distribution of oxygenated blood to several vital organs, which reduces the blood flow to the intestinal tract, decreases mucous production, and shrinks gut barrier integrity [47]. Integrative analyzes of endurance athletes, such as cyclists, have shown enrichment of metabolic pathways involved in oxidative energy production and SCFA biosynthesis, which suggests that endurance training selectively shapes microbial functions that are linked to energy metabolism [18]. Accordingly, it can be hypothesized that increased gut permeability among athletes with higher performance levels might have contributed to the mentioned variations between the present study and the aforementioned literature. Furthermore, it is important to consider that various types of training and competitions, due to their static or dynamic properties, show diverse discrete impacts on the gut microbial community [62]. Herein, middle‐distance runners possessed higher levels of Ε‐proteobacteria and *Veillonella* spp., while Firmicutes, Actinobacteria, and *M. smithii* were more abundant among speed runners.

Among the investigated taxa, *A. muciniphila* was the most abundant species among speed runners, which may reflect a sport‐specific microbial adaptation, particularly in response to the intense, glycolytic demands of sprint training. Recent metagenomic analyzes by Aya et al. [18] revealed that anaerobic athletes exhibit enrichment of microbial pathways that are linked to mucin degradation, epithelial glucose signaling, and energy flux through glycolysis. Similarly, Cheng et al. [10] identified host‐microbiome co‐adaptive genomic signatures in high‐intensity athletes, including the upregulation of genes involved in carbohydrate utilization and mucosal integrity. As a mucin‐degrading symbiont, *A. muciniphila* is known to thrive in environments of accelerated mucosal turnover to modulate glucose homeostasis and metabolic efficiency. Accordingly, the high level of *A. muciniphila* in sprinters may potentially lead to preferential mucosal adaptation in response to repeated glycolytic stress and epithelial remodeling. In addition, a meta‐analysis showed that probiotic supplementation significantly improved exercise recovery and reduced markers of muscle damage, such as creatine kinase, which reflect a potential supportive role for gut modulation in postexercise repair processes [21]. Bressa et al. [64] reported that women with regular exercise habits had a substantial number of health‐promoting microbiota, such as *A. muciniphila*. Indeed, this bacterium refines intestinal penetrance and enhances inflammatory responses as well as insulin resistance and fat accumulation [65, 66]. Nevertheless, unendurable and tiring endurance training increases colonic transit time and causes inflammation, as explained [39]. The increase in *F. nucleatum* in response to middle‐distance and endurance running is noteworthy as this group of bacteria includes several potentially inflammatory and disease‐causing features that are linked to gut barrier impairment [67]. All in all, further investigations are required to identify the explicit mechanism of action of aforesaid bacterial groups on the quality of performance among runners of various fields.

## 5 Conclusion

**Figure 8:**
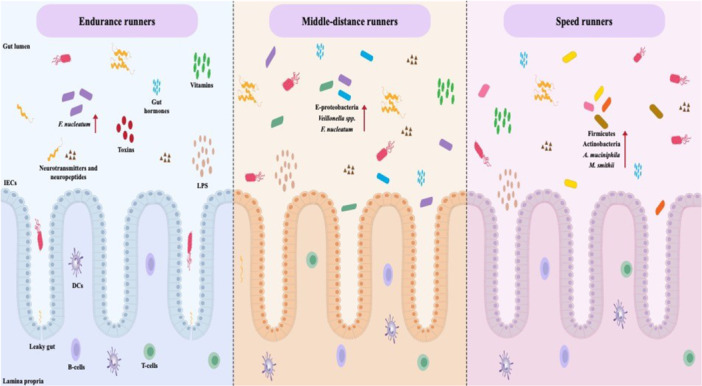
This figure summarizes key microbial taxa associated with endurance, middle‐distance, and speed runners as identified in this study. Each athletic discipline is linked to distinct gut commensals with potential roles in energy metabolism, immune modulation, and intestinal homeostasis. Arrows denote the increased or decreased abundance of selected microbial taxa. Taxa such as *A. muciniphila* and *F. prausnitzii* may support mucosal integrity and exert anti‐inflammatory effects, while elevated levels of *F. nucleatum* in endurance runners may indicate gut barrier disruption. Athlete‐associated bacteria may modulate neurotransmitter and vitamin production from dietary components, influence cytokine secretion, regulate immune cell function, and contribute to the activation of autoreactive immune cells. Intense endurance training may disrupt intestinal homeostasis, leading to gut dysbiosis, reduced SCFA production, impaired immune regulation, decreased microbial diversity, and an increase in proinflammatory taxa such as *F. nucleatum*. DCs, dendritic cells; LPS, lipopolysaccharides; IECs, intestinal epithelial cells.

This study illuminates the variations in the relative abundance of a selection of gut microbiota among Iranian semi‐professional and professional runners across different running disciplines (Figure 8). The distinct microbial taxa observed in speed, middle‐distance, and endurance runners corroborate the relationship between running disciplines and the gut microbiota composition, as well as their impact on performance level. These findings contribute to our understanding of how athletic training influences the gut microbiome, highlighting the potential for microbiota to be a factor in optimizing athletic performance. Of note, because of the limited accessibility to athletes participating in Tokyo 2020 Olympics, the P3 subgroup remained unpopulated. Given the cross‐sectional design and limited cohort size, future studies should involve larger longitudinal cohorts and comprehensive sequencing techniques, such as 16S rRNA gene profiling or shotgun metagenomics for runner's microbiota, to expand upon these findings. Furthermore, evaluating the functional characteristics of athletes' gut microbiome is crucial for gaining a more comprehensive understanding of how the athletic microbiota signatures influence performance metrics. To further enhance our understanding, future research should aim to address the effects of sustained training on the gut microbiota to provide deeper insights into the adaptive responses of the microbiome to different forms and intensities of exercise.

## Author Contributions

**Hiwa Nazari:** methodology, investigation, writing – original draft, formal analysis, software. **Armitasadat Emami Meibodi:** data curation, writing – original draft. **Minoo Bassami:** conceptualization, methodology, validation, formal analysis, supervision, project administration, writing – review and editing, data curation. **Meysam Olfatifar:** formal analysis, software. **Abbas Yadegar:** conceptualization, methodology, data curation, validation, formal analysis, supervision, resources, project administration, visualization, funding acquisition, writing – review and editing.

## Conflicts of Interest

The authors declare that they have no conflicts of interest. All authors have read and approved the final version of the manuscript. The corresponding author had full access to all of the data in this study and takes complete responsibility for the integrity of the data and the accuracy of the data analysis.

## Transparency Statement

The lead author Minoo Bassami, Abbas Yadegar affirms that this manuscript is an honest, accurate, and transparent account of the study being reported; that no important aspects of the study have been omitted; and that any discrepancies from the study as planned (and, if relevant, registered) have been explained.

## Supporting information


**TABLE S1:** Individual data on dietary habits, alcohol use, and training volume in hours per week are presented for speed runners.

**TABLE S2:** Individual data on dietary habits, alcohol use, and training volume in hours per week are presented for middle‐distance runners.

**TABLE S3:** Individual data on dietary habits, alcohol use, and training volume in hours per week are presented for endurance runners.

**TABLE S4:** Correlation coefficients, raw P values, 95% confidence intervals, and FDR‐adjusted P values for each microbial taxon.

## Data Availability

The authors confirm that the data supporting the findings of this study are available within the article and its supporting materials.
