# Supplementary material for: Association of Gut Microbiota With Performance Level Among Iranian Professional and Semi‐Professional Runners: A Cross‐Sectional Study
Source: Health Sci Rep. 2025 Oct 3;8(10):e71319. doi: 10.1002/hsr2.71319 (PMC12491851; doi:10.1002/hsr2.71319)
Supplement: Supplementary file 2 — TABLE S2: Individual data on dietary habits, alcohol use, and training volume in hours per week are presented for middle‐distance runners. [file HSR2-8-e71319-s003.docx]

| Runners | Diet | Alcohol consumption (per week) | Exercise load (hours/week) |
| --- | --- | --- | --- |
| Sp1 | Equal protein, fat, carbs | 0 | 11-15 |
| Sp2 | Equal protein, fat, carbs | 0 | 6-10 |
| Sp3 | High complex carbs | 0 | 6-10 |
| Sp4 | Equal protein, fat, carbs | 0 | 6-10 |
| Sp5 | High complex carbs | 0 | 6-10 |
| Sp6 | High complex carbs | 1-3 | 11-15 |
| Sp7 | High complex carbs | 0 | 16-20 |
| Sp8 | High complex carbs | 0 | 11-15 |
| Sp9 | Equal protein, fat, carbs | 0 | 6-10 |
| Sp10 | High complex carbs | 0 | 6-10 |
| S1 | Vegetarian | 0 | 6-10 |
| S2 | Equal protein, fat, carbs | 0 | 11-15 |
| S3 | High complex carbs | 1-3 | 11-15 |
| S4 | High complex carbs | 0 | 16-20 |
| S5 | High complex carbs | 0 | 11-15 |
| S6 | Equal protein, fat, carbs | 0 | 11-15 |
| S7 | High complex carbs | 0 | 16-20 |
| S8 | High complex carbs | 0 | 6-10 |
| S9 | Equal protein, fat, carbs | 0 | 11-15 |
| S10 | High complex carbs | 0 | 11-15 |

**T A B L E S2** Individual data on dietary habits, alcohol use, and training volume in hours per week are presented for middle-distance runners. Dietary classification is based on reported food intake, as recorded in structured dietary questionnaires.
