# Supplementary material for: Association of Gut Microbiota With Performance Level Among Iranian Professional and Semi‐Professional Runners: A Cross‐Sectional Study
Source: Health Sci Rep. 2025 Oct 3;8(10):e71319. doi: 10.1002/hsr2.71319 (PMC12491851; doi:10.1002/hsr2.71319)
Supplement: Supplementary file 3 — TABLE S3: Individual data on dietary habits, alcohol use, and training volume in hours per week are presented for endurance runners. [file HSR2-8-e71319-s002.docx]

| Runners | Diet | Alcohol consumption (per week) | Exercise load (hours/week) |
| --- | --- | --- | --- |
| Sp1 | High complex carbs | 0 | 11-15 |
| Sp2 | Gluten-free | 0 | 16-20 |
| Sp3 | High complex carbs | 1-3 | 11-15 |
| Sp4 | Equal protein, fat, carbs | 0 | 11-15 |
| Sp5 | High complex carbs | 0 | 6-10 |
| Sp6 | High complex carbs | 0 | 11-15 |
| Sp7 | High complex carbs | 0 | 11-15 |
| Sp8 | High complex carbs | 0 | 11-15 |
| Sp9 | Equal protein, fat, carbs | 0 | 6-10 |
| Sp10 | High complex carbs | 0 | 11-15 |
| S1 | Vegetarian | 0 | +20 |
| S2 | Equal protein, fat, carbs | 0 | +20 |
| S3 | High complex carbs | 0 | 11-15 |
| S4 | High complex carbs | 0 | +20 |
| S5 | High complex carbs | 0 | 11-15 |
| S6 | High complex carbs | 0 | 16-20 |
| S7 | High complex carbs | 0 | +20 |
| S8 | High complex carbs | 0 | 16-20 |
| S9 | High complex carbs | 0 | +20 |
| S10 | High complex carbs | 0 | 16-20 |

**T A B L E S3** Individual data on dietary habits, alcohol use, and training volume in hours per week are presented for endurance runners. Dietary classification is based on reported food intake, as recorded in structured dietary questionnaires.
