# Supplementary material for: Association of Gut Microbiota With Performance Level Among Iranian Professional and Semi‐Professional Runners: A Cross‐Sectional Study
Source: Health Sci Rep. 2025 Oct 3;8(10):e71319. doi: 10.1002/hsr2.71319 (PMC12491851; doi:10.1002/hsr2.71319)
Supplement: Supplementary file 4 — TABLE S4: Correlation coefficients, raw P values, 95% confidence intervals, and FDR‐adjusted P values for each microbial taxon. [file HSR2-8-e71319-s004.docx]

**T A B L E S4** Correlation coefficients, raw *P* values, 95% confidence intervals, and FDR-adjusted *P* values for each microbial taxon. The table represents association between the relative abundance of selected microbial taxa and the performance level of runners. Adjusted *P* values are presented to reflect potential microbiome-performance associations.

| **Variable** | **Spearman’s rho** | ***P* value** | **CI-lower** | **CI-upper** | **FDR-adjusted *P* value** |
| --- | --- | --- | --- | --- | --- |
| Firmicutetes | -0.401546457 | 0.001473148 | -0.598631179 | -0.165487879 | 0.00220972 |
| Actinobacteria | -0.69613639 | 6.61E-10 | -0.775407909 | -0.59247527 | 3.97E-09 |
| Enterobacteriaceae | -0.551432855 | 4.96E-06 | -0.682675695 | -0.386122552 | 9.93E-06 |
| *Bifidobacterium* spp. | -0.343551432 | 0.007199081 | -0.56310923 | -0.061433972 | 0.008638897 |
| *FaecalBacterium prausnitzii* | -0.578370935 | 1.3E-06 | -0.705206004 | -0.341172134 | 3.9E-06 |
| *Methanobrevibacter smithii* | 0.269713767 | 0.037156646 | 0.032371217 | 0.47076751 | 0.037156646 |
